# Extensive abdominal postsurgical Pyoderma Gangrenosum

**DOI:** 10.1002/ccr3.6673

**Published:** 2022-12-05

**Authors:** Raquel Lalanda

**Affiliations:** ^1^ General Surgery Centro Hospitalar do Médio Tejo (CHMT) Tomar Portugal

**Keywords:** dermatosis, gangrenosum, pathergy, postoperative, pyoderma, ulcerative

## Abstract

We highlight the key aspects for the diagnosis of Postoperative pyoderma gangrenosum: “Pathergy phenomenon” at surgical site incisions; “Skip lesions”: central venous catheter site lesions were similar to abdominal wall lesions despite distance between them; Lack of response to antibiotics or debridement; Immediate response to corticosteroid therapy.

## 1 CASE PRESENTATION

**FIGURE 1:**
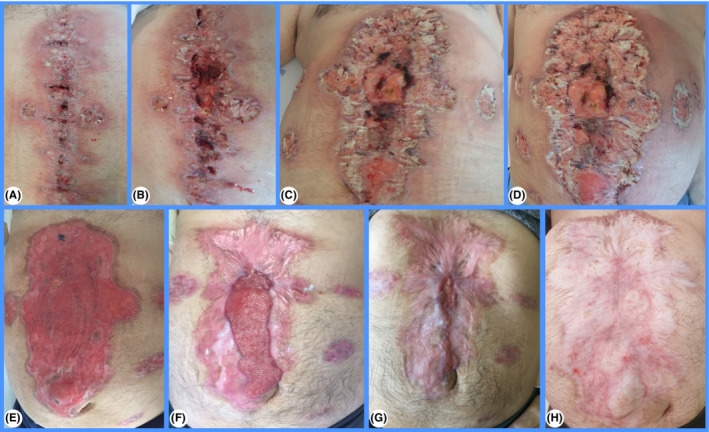
Laparotomy and laparoscopy sites on 16th, 17th, 20th, and 21th post‐operative day (A–D). Laparotomy and laparoscopy sites 6, 7, 8, and 30 months later (E–H)

**FIGURE 2:**
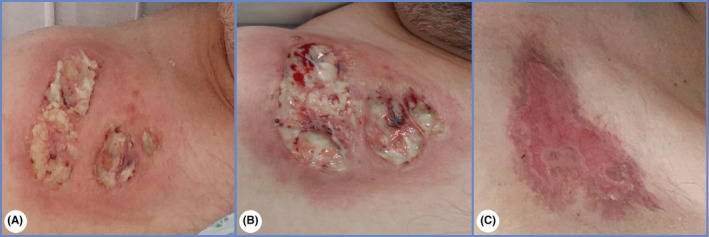
Central venous catheter site on 16th and 20th post‐operative day (A,B). Central venous catheter site 6 months later (C)

A 50‐year‐old man underwent a laparoscopic Nissen fundoplication converted to open surgery due to visceral obesity. He was discharged 3 days later without immediate postoperative complications. His past medical history included diabetes mellitus and ulcerative colitis without symptoms or previous treatment. Patient presented 13 days post‐operatively with advancing erythema over the midline laparotomy incision and laparoscopic access sites. He was hemodynamically stable and afebrile. Surgical site infection was suspected. Patient refused hospitalization and was discharged against medical advice with a course of oral antibiotics. He returned 3 days later with extensive erythema and skin ulceration around necrotic centers with purulent debris over the surgical incision sites (Figure 1). Investigations revealed raised inflammatory markers with normal electrolytes, renal and liver function tests. Wound cultures were positive for Enterococcus faecalis sensitive to ampicillin, but blood cultures remained persistently negative. Neutrophilic infiltrates were identified on microscopy. Despite immediate wound care with bedside debridement and intravenous antibiotic therapy, lesions spread centrifugally. Patient deteriorated clinically, developed fever, hypotension, and tachycardia. Similar lesions also appeared over the subclavian central venous catheter previous site (Figure 2).

## 2 QUIZ QUESTION: WHAT IS YOUR DIAGNOSIS?

Postoperative pyoderma gangrenosum (POPG) was suspected. A multidisciplinary team of surgeons, gastroenterologists, and dermatologists decided to start him on a trial of high dose pulse intravenous steroids with 1 g of methylprednisolone given daily for 5 days. An immediate stabilization of the skin ulcers occurred. Clinical and serological improvements were also observed. Patient was switched to oral corticoids which were tapered down and antibiotic therapy was stopped. Lesions progressively began to present signs of granulation, and he was discharged 55 days later. A gastroenterologist appointment was scheduled. Ulcers were healed at six‐month follow‐up (Figure 1 and Figure 2).

Pyoderma gangrenosum (PG) is a rare inflammatory neutrophilic dermatosis^1^ characterized by ulcerative skin eruptions with exudative violet edges, central necrotic areas, and an erythematous halo^2^ of the surrounding skin. Inflammatory bowel disease is present in 17.6% of cases, with ulcerative colitis being the most frequent.^1^ Pathergy phenomenon after skin trauma or surgical incisions was responsible for POPG.^2^, ^3^ POPG can present after a variable period of time ranging from 4 days–6 weeks.^1^, ^2^ It is often misdiagnosed as surgical site infection,^1^, ^2^ one of the most frequent postoperative complications, because of positive wound swabbing.^1^, ^2^

Diagnostic criteria for PG were proposed and included: rapid progression of cutaneous, painful, necrotic ulcers, with irregular violet edge; exclusion of other causes of cutaneous ulceration; previous history suggestive of pathergy; systemic diseases associated with PG; histological samples compatible with PG; response to corticosteroid therapy.^2^, ^3^

This case report meets the criteria described above.

The patient presented with a painful, necrotic ulcer affecting the entire anterior abdominal wall. He had increased inflammatory parameters and persistent fever, which led to the initial diagnosis of surgical site infection. Debridement and intravenous antibiotic therapy did not improve disease progression. The initiation of immunosuppression with corticosteroid therapy after the proposal for the diagnosis of POPG was a bold decision. POPG is a rare disease that represents a diagnostic and therapeutic challenge.

## AUTHOR CONTRIBUTION

Raquel Lalanda performed the research; analyzed the data; and wrote the paper.

## CONFLICT OF INTEREST

The author has read and approved the final manuscript. The author declare no conflict of interests.

## CONSENT

Written informed consent was obtained from the patient to publish this case report in accordance with the journal's patient consent policy.

## Data Availability

The data that support the findings of this study are available from the corresponding author upon reasonable request.
